# Photoexcited Properties of Tin Sulfide Nanosheet-Decorated ZnO Nanorod Heterostructures

**DOI:** 10.1186/s11671-017-2022-z

**Published:** 2017-04-07

**Authors:** Yuan-Chang Liang, Tsai-Wen Lung, Nian-Cih Xu

**Affiliations:** Institute of Materials Engineering, National Taiwan Ocean University, Keelung, 20224 Taiwan

**Keywords:** Morphology, Surface, Sulfide, Sputtering, Heterostructure

## Abstract

In this study, ZnO–Sn_2_S_3_ core–shell nanorod heterostructures were synthesized by sputtering Sn_2_S_3_ shell layers onto ZnO rods. The Sn_2_S_3_ shell layers consisted of sheet-like crystallites. A structural analysis revealed that the ZnO–Sn_2_S_3_ core–shell nanorod heterostructures were highly crystalline. In comparison with ZnO nanorods, the ZnO–Sn_2_S_3_ nanorods exhibited a broadened optical absorption edge that extended to the visible light region. The ZnO–Sn_2_S_3_ nanorods exhibited substantial visible photodegradation efficiency of methylene blue organic dyes and high photoelectrochemical performance under light illumination. The unique three-dimensional sheet-like Sn_2_S_3_ crystallites resulted in the high light-harvesting efficiency of the nanorod heterostructures. Moreover, the efficient spatial separation of photoexcited carriers, attributable to the band alignment between ZnO and Sn_2_S_3_, accounted for the superior photocatalytic and photoelectrochemical properties of the ZnO–Sn_2_S_3_ core–shell nanorod heterostructures.

## Background

A considerable number of researchers have examined the solar light-harvesting characteristics of oxide semiconductors to solve energy and environmental problems. Because of their high specific surface area values, nanostructured oxide semiconductors can be applied with high efficiency in various photoexcited devices [1–3]. Among various oxides, ZnO is a promising semiconductor material with a wide bandgap of approximately 3.3 eV, a large exciton binding energy of 60 meV, stable chemical properties, high electrochemical activity, and low cost. Thus, researchers have focused on the utilization of one-dimensional ZnO nanostructures in abundant photovoltaic, optoelectronic, and energy-related device applications [4–6]. However, the wide bandgap of ZnO significantly restricts its use under solar light because only a limited percentage of solar radiation is within the ultraviolet (UV) range. Several methods, such as crystal defect density control and chemical composition modification of ZnO nanostructures, have been adopted to improve the solar light-harvesting efficiency of ZnO [7, 8] and have produced limited improvements of the light-harvesting efficiency of ZnO. The efficiency levels of photoexcited devices made from ZnO nanostructures under solar light irradiation still require substantial improvement.

Improvement of the light-harvesting efficiency of one-dimensional ZnO oxides and reduction of the recombination rate of photoexcited carriers in these oxides are crucial to produce various highly efficient photoexcited nanodevices from ZnO nanostructures. Research has proven the construction of one-dimensional ZnO-based heterostructures to be a promising approach to obtain ZnO nanostructures with relatively high photoactivated performance; by contrast, devices with single ZnO components are less promising. Recent studies have reported that one-dimensional semiconductor composites are of potential for photocatalytic applications [9–11]. Moreover, ZnO nanostructures coupled with narrow-bandgap materials, such as CdS and CdSe, exhibit enhanced photoactivated performance levels [12, 13]. Thus, it is highly desirable to design and fabricate one-dimensional ZnO-based heterostructures through appropriate band alignment and coupling with narrow-bandgap semiconductors for applications in various photoexcited devices. The metal sulfides of semiconductors usually exhibit superior light absorption in the visible region and exhibit absorption edges within the near-infrared region, which facilitate their response to the visible light of the solar spectrum, thus improving light-harvesting efficiency. Moreover, metal sulfides such as SnS, SnS_2_, and Sn_2_S_3_ have been used for visible light-driven photocatalytic reactions [14–16]. Among various metal sulfides, Sn_2_S_3_ has a narrow bandgap of approximately 1.1–2.05 eV and exhibits superior optical properties [16–18]. It is nonpoisonous, chemically stable, and inexpensive; therefore, it is a potential visible light sensitizer for photocatalytic, photovoltaic, and photoelectric devices. However, few studies have focused on coupling Sn_2_S_3_ with ZnO to form low-dimensional heterostructures. In the present study, ZnO–Sn_2_S_3_ core–shell heterostructures with special three-dimensional shell architecture were synthesized by sputtering Sn_2_S_3_ crystallites onto the surfaces of ZnO nanorods; the superior photoactivated properties of the ZnO–Sn_2_S_3_ core–shell heterostructures were investigated and were found to be highly correlated with the microstructures of the heterostructures.

## Methods

In this study, ZnO-based core–shell nanorod heterostructures with the Sn_2_S_3_ shell layer (ZnO–Sn_2_S_3_ nanorods) were synthesized through a combinational methodology of hydrothermal and sputtering. Hydrothermally synthesized high-density ZnO nanorods were used as templates for growing the ZnO–Sn_2_S_3_ nanorod heterostructures. Detailed experiments on the hydrothermal synthesis of ZnO nanorods have been described elsewhere [19]. The Sn_2_S_3_ shell layers were fabricated by using radio frequency magnetron sputtering. The target sputtering power was fixed at 40 W in pure Ar ambient. The thin-film growth temperature of the Sn_2_S_3_ thin films was maintained at 250 °C. During thin-film sputtering deposition, the gas pressures of the Sn_2_S_3_ shell layers were fixed at 10 mTorr.

Sample crystal structures were investigated by X-ray diffraction (XRD; Bruker D2 PHASER) using Cu Kα radiation. The surface morphology of the samples was investigated by scanning electron microscopy (SEM; Hitachi S-4800). The microstructures of the rod samples were characterized by high-resolution transmission electron microscopy (HRTEM; Philips Tecnai F20 G2). The optical absorption spectra of the samples were recorded in the wavelength range of 300–800 nm by using UV–Vis spectrophotometer (Jasco V750). Room temperature-dependent photoluminescence (PL; Horiba HR800) spectra were obtained using the 325 nm line of a He–Cd laser. The photocatalytic activities of as-prepared samples were investigated by measuring the photodegradation rate of methylene blue (MB; 10^−6^ M) as organic dyes under solar light irradiation. Prior to illumination, MB aqueous solution containing different samples were continuously stirred in the dark for 30 min to achieve adsorption/desorption equilibrium between the catalysts. After reaction, the solution was analyzed by measuring the absorption intensity of the main peak at approximately 663 nm by UV–Vis spectrophotometer. The photoelectrochemical (PEC) properties were measured in a convenient three electrodes electrochemical system (SP-50 Potentiostat/Galvanostat). The pure ZnO and ZnO–Sn_2_S_3_ nanorod heterostructures grown on the conductive F-doped SnO_2_ glasses were used as the working electrode, a platinum wire as the counter electrode, and an Ag/AgCl as the reference electrode. Aqueous solution containing 0.25 M of Na_2_S and 0.35 M of Na_2_SO_3_ was utilized as the electrolyte in an electrochemical system [20]. The intensity of illumination at the sample position was fixed to be approximately 50 mW cm^−2^ in this study.

## Results and Discussion

**Fig. 1:**
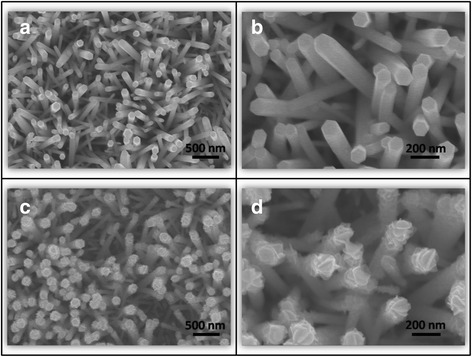
**a** Low-magnification SEM micrograph of ZnO nanorods. **b** High-magnification SEM micrograph of ZnO nanorods. **c** Low-magnification SEM micrograph of ZnO–Sn_2_S_3_ nanorods. **d** High-magnification SEM micrograph of ZnO–Sn_2_S_3_ nanorods

Figure 1a, b illustrates the morphology of the ZnO nanorods. The surfaces of the ZnO nanorods were smooth with a hexagonal crystal feature. Figure 1c, d depicts the morphologies of the ZnO–Sn_2_S_3_ core–shell nanorods. SEM micrographs demonstrated that the hexagonal ZnO nanorods became circular, and the surfaces of the ZnO–Sn_2_S_3_ nanorods exhibited undualations and a visible sheet-like crystal texture. The sheet-like crystallites on the surfaces of the ZnO–Sn_2_S_3_ core–shell nanorods had sharp peripheries and were homogeneously distributed on the ZnO nanorods. The SEM micrographs showed that the surfaces of the ZnO–Sn_2_S_3_ nanorods were rougher than those of the ZnO nanorods, thus the ZnO–Sn_2_S_3_ nanorods had larger surface areas.

**Fig. 2:**
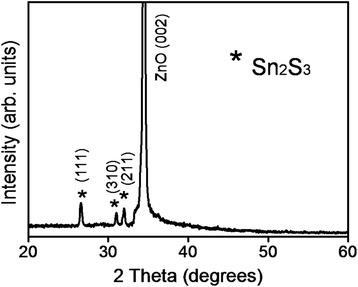
XRD pattern of ZnO–Sn_2_S_3_ nanorods

Figure 2 shows the XRD pattern of the ZnO–Sn_2_S_3_ nanorods. The XRD pattern displays an intense and sharp Bragg reflection centered at approximately 34.4°, which is ascribed to the (002) crystallographic plane of the hexagonal ZnO nanorods (JCPDS no. 05-0664). The ZnO nanorods were highly crystalline, and the grains were oriented along the *c*-axis. Moreover, the XRD pattern displays three other sharp Bragg reflections with relatively low intensities centered at approximately 26.5°, 30.8°, and 31.9°. These Bragg reflections originated from the (111), (310), and (211) crystallographic planes of the orthorhombic Sn_2_S_3_ (JCPDS no. 14-0619). The XRD pattern indicates that the as-deposited Sn_2_S_3_ shell layer was polycrystalline, and the as-synthesized ZnO–Sn_2_S_3_ nanorods were highly crystalline.

**Fig. 3:**
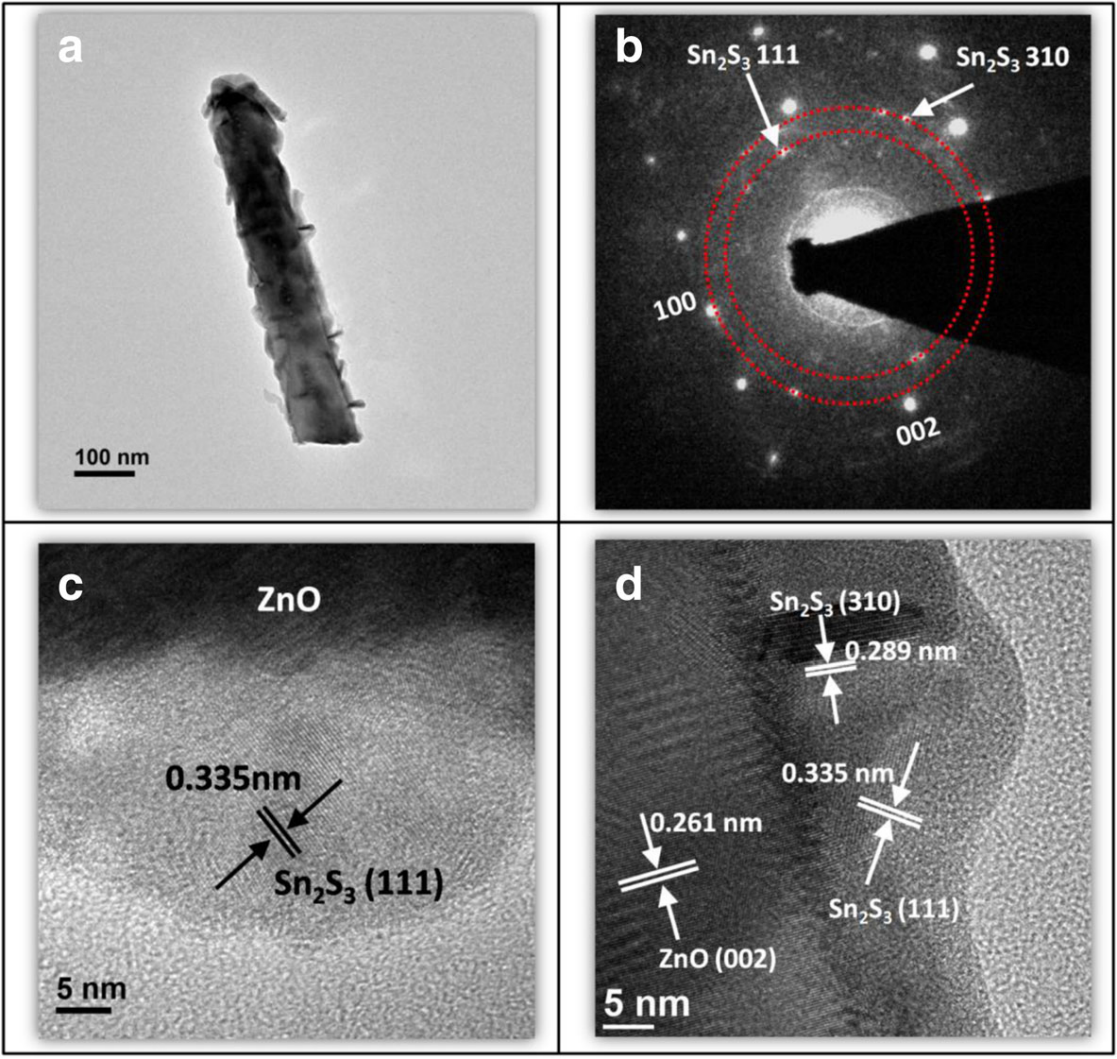
TEM analyses of the ZnO–Sn_2_S_3_ nanorod heterostructure. **a** Low-magnification TEM image of the ZnO–Sn_2_S_3_ nanorod. **b** SAED pattern of the nanorod. **c**, **d** HRTEM images taken from the local regions of the nanorod

Figure 3a shows a low-magnification TEM image of the ZnO–Sn_2_S_3_ core–shell nanorod. The TEM image reveals that the ZnO–Sn_2_S_3_ nanorod had sheet-like surface morphology, which was considerably rough. Figure 3b shows the selected area electron diffraction (SAED) pattern detected from the nanorod. The SAED pattern exhibits one set of diffraction spots that can be indexed into the [010] zone axis of the hexagonal ZnO nanorod and several diffraction rings that can be indexed into Sn_2_S_3_ (111) and (310) crystallographic planes. The SAED pattern reveals that the ZnO–Sn_2_S_3_ core–shell nanorod exhibited appropriate crystalline phases and that the crystallographic planes were in close agreement with the XRD results. Figure 3c, d presents HRTEM images taken from the outer regions of the ZnO–Sn_2_S_3_ nanorod. The HRTEM images reveal clear and long-range ordered lattice fringes that were regionally distributed. The lattice fringes with intervals of approximately 0.261 nm in the inner region corresponded to hexagonal ZnO (002). The lattice fringes with intervals of approximately 0.289 and 0.335 nm in the outer region matched the spacing distances of Sn_2_S_3_ (310) and (111) crystallographic planes, respectively. The TEM analyses reveal a highly crystalline ZnO–Sn_2_S_3_ core–shell nanorod with a strongly undulated surface.

**Fig. 4:**
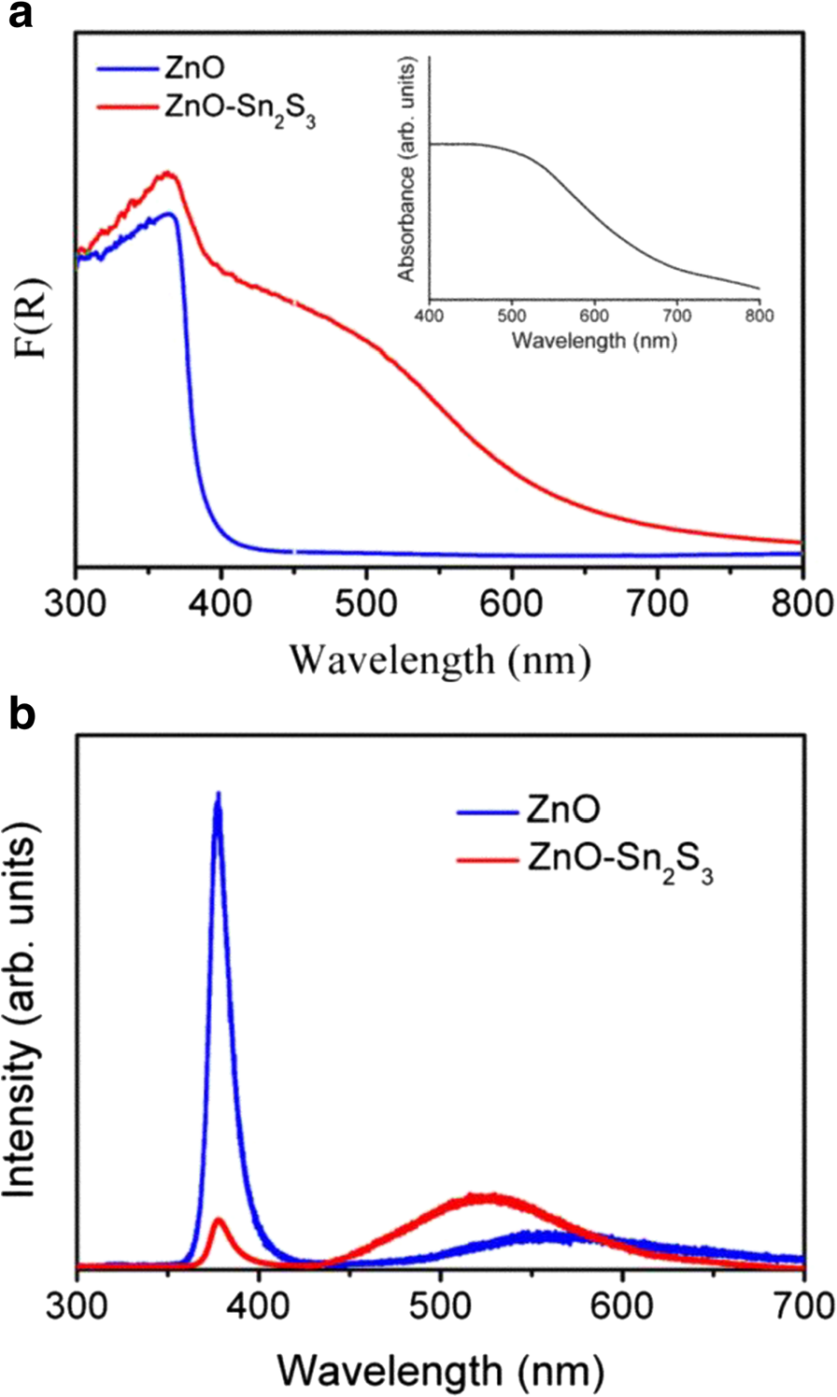
**a** Optical absorbance spectra of the ZnO and ZnO–Sn_2_S_3_ nanorods. The *inset* shows the optical absorbance spectrum of the Sn_2_S_3_ film. **b** PL spectra of the ZnO and ZnO–Sn_2_S_3_ nanorods

The optical absorption edges of the pure ZnO and ZnO–Sn_2_S_3_ rods were evaluated by measuring the diffuse reflectance spectra of the samples and by converting these spectra into absorption coefficient spectra with the Kubelka–Munk function [21, 22]. Figure 4a displays the Kubelka–Munk conversion spectra of the pure ZnO and ZnO–Sn_2_S_3_ nanorods. Typically, ZnO has a wide-bandgap value in the UV light region. Moreover, Sn_2_S_3_ with the same effective shell thickness showed a bandgap value in the visible light region as exhibited in the inset of Fig. 4a; the bandgap value can be estimated from the absorbance spectra by using the Tauc plot and the bandgap is approximately 1.83 eV. In this study, in comparison with the pure ZnO nanorods, the ZnO–Sn_2_S_3_ nanorods exhibited a broadened optical absorption edge that extended to the visible light region. The ZnO–Sn_2_S_3_ nanorods exhibited visible light-sensitive features. The broader absorption spectrum and lower absorption edge energy indicated that the ZnO–Sn_2_S_3_ nanorod heterostructures had superior optical absorption capability under light illumination. It has been reported that the construction of a heterostructure by coupling with the visible light sensitizers similarly extended the optical absorption edge of the wide-bandgap oxides in other material systems (i.e., ZnO–CdS, TiO_2_–Ag_2_S, and SnO_2_–Fe_2_O_3_) [23–25]. Figure 4b displays the room-temperature PL spectra of the ZnO and ZnO–Sn_2_S_3_ nanorods. A UV emission band centered at approximately 378 nm can be ascribed to the near-band edge emission of the ZnO rods [19]. Furthermore, the ZnO and ZnO–Sn_2_S_3_ nanorods exhibited broad emission bands centered at approximately 560 and 530 nm, respectively. These broad visible light emission bands are referred to as deep-level or trap-state emission bands and might be associated with structural defects arising from the oxygen vacancies of the ZnO core and the shallow trap caused by the surface states of the sulfide shell layer [19, 26]. In comparison with the broad emission bands, the intensity of the near-band edge emission from ZnO was markedly quenched when the Sn_2_S_3_ crystallites were sputtered onto the surfaces of the ZnO nanorods. This finding indicated that the recombination of the photogenerated charge carrier was considerably inhibited in the ZnO–Sn_2_S_3_ heterostructures.

**Fig. 5:**
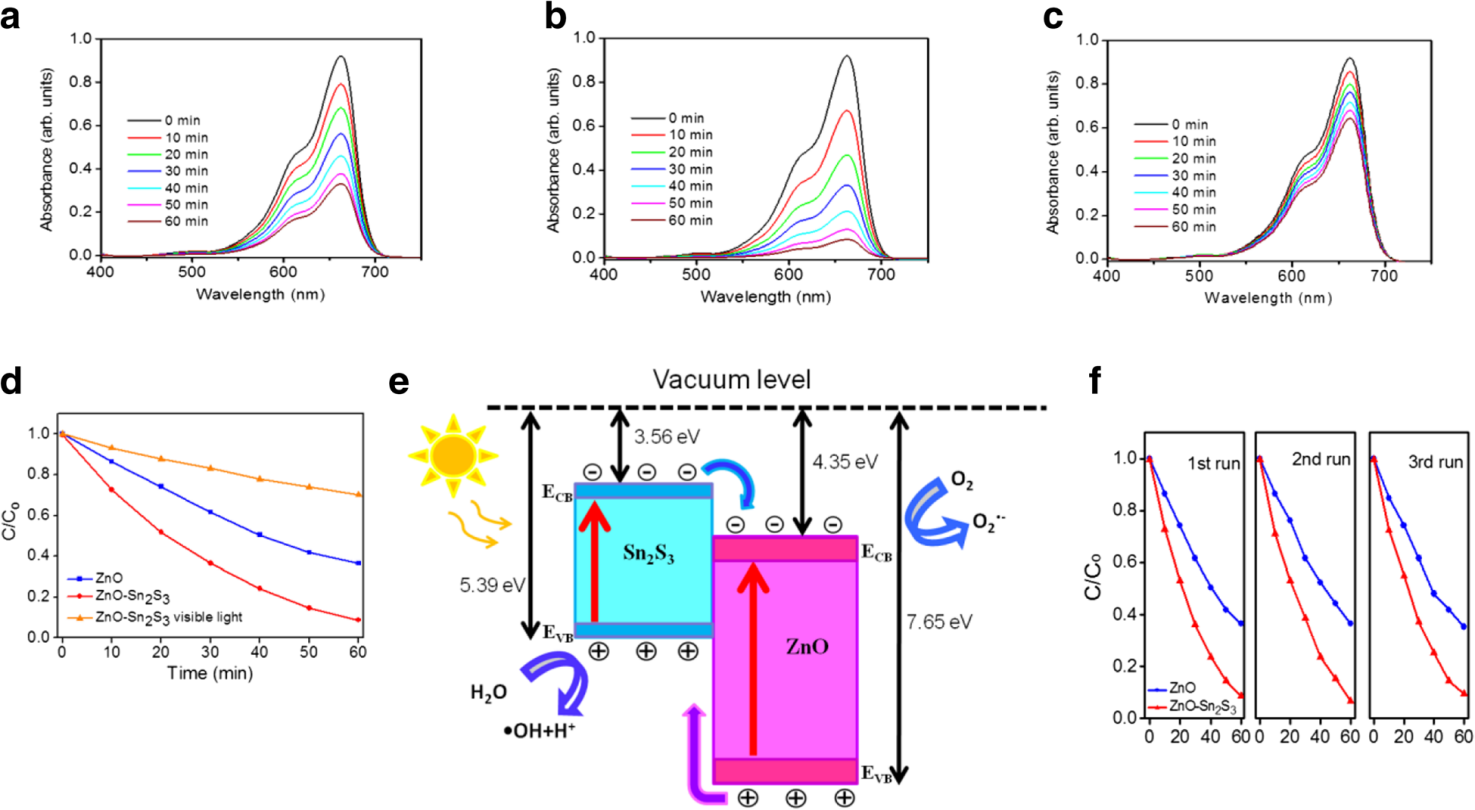
Intensity variation of absorbance spectra of MB solution vs. degradation duration containing various nanorods samples under light illumination. **a** Pure ZnO nanorods under solar light illumination. **b** ZnO–Sn_2_S_3_ nanorods under solar light illumination. **c** ZnO–Sn_2_S_3_ nanorods under visible light illumination. **d** *C*/*C*_o_ vs. irradiation time curves for MB solution with various nanorod samples under light illumination. **e** A schematic of band alignment and charges transfer of the ZnO–Sn_2_S_3_ heterostructure under light illumination. **f** Recycled performance (three test runs) in the presence of ZnO–Sn_2_S_3_ nanorods for photodegradation of MB dyes

The photocatalytic activities of the nanorod samples were evaluated by photodegradation of MB under solar light irradiation with various durations. Figure 5a, b illustrates the time course-dependent absorbance spectra of the ZnO and ZnO–Sn_2_S_3_ nanorods under solar light illumination. The visible and intense peaks of the absorption spectra at approximately 663 nm can be ascribed to monomeric MB. The intensity of the absorbance peak centered at approximately 663 nm for the MB solution containing the ZnO and ZnO–Sn_2_S_3_ nanorods decreased with increased reaction duration. The photodegradation size was defined as (*C*/*C*_o_), where *C*_o_ is the concentration of aqueous MB without irradiation after dark adsorption equilibrium, and *C* is the concentration of aqueous MB corresponding to a given light irradiation duration [27]. For a comparison, the absorbance spectra of the MB solution containing ZnO–Sn_2_S_3_ nanorods under visible light irradiation with various durations were also shown in Fig. 5c. An ultraviolet light filter was used during the photodegradation test. Figure 5d shows that under light illumination, the photodegradation size of MB increased with reaction duration when ZnO nanorods were used as catalysts. In comparison with the ZnO nanorods, the ZnO–Sn_2_S_3_ nanorods photodegraded MB organic dyes with substantially higher efficiency under the same reaction conditions. The superior photocatalytic performance of the ZnO–Sn_2_S_3_ core–shell structure is attributable to the band relation of the heterostructure. Notably, Sn_2_S_3_ is a narrow-bandgap semiconductor with a reported electron affinity of approximately 3.56 eV [17], whereas ZnO is an n-type wide-bandgap semiconductor with a reported electron affinity of 4.35 eV [28]. These data may provide reliable references to approximately estimate the relative band edge positions of the two semiconductors. The contact of ZnO and Sn_2_S_3_ forms a type II band alignment structure. When the ZnO–Sn_2_S_3_ heterojunction is formed, electrons tend to flow from Sn_2_S_3_ to ZnO, resulting in electron accumulation on the ZnO side [29]. Figure 5e illustrates the possible band alignment of the ZnO–Sn_2_S_3_ heterostructure. As shown in Fig. 5e, when the ZnO–Sn_2_S_3_ nanorods are irradiated by solar light, the ZnO cores of the heterostructures respond to UV light because of the wide bandgap of ZnO, and numerous photoexcited electrons and holes are generated. Simultaneously, photoexcited electron–holes are also generated in Sn_2_S_3_ shells under light irradiation. The photoexcited electrons in the Sn_2_S_3_ shell layers tend to be transferred from the conduction band of Sn_2_S_3_ to the conduction band of ZnO through the interface, whereas the photoexcited holes in the ZnO are transferred to the valence band of Sn_2_S_3_. A predictable photoexcited charge transfer between ZnO and Sn_2_S_3_ can be noted. A previous study demonstrated that efficient spatial charge separation prolongs the lifetime of photoexcited charges in a semiconductor composite system [22]. In that study, a higher-than-usual number of photoexcited electrons near the surfaces of ZnO rods were captured by O_2_ molecules to yield superoxide radical anions (O_2_^·−^) and hydrogen peroxide (H_2_O_2_); subsequently, the reaction of O_2_^·−^ with H_2_O_2_ generated ·OH. Moreover, a higher-than-usual number of photoexcited holes near the shell surfaces of the rod heterostructures oxidized H_2_O molecules to produce hydroxyl radicals (·OH), which were strong oxidizing agents that effectively decomposed MB [22]. The band configuration of the ZnO–Sn_2_S_3_ heterostructure reduces the electron–hole recombination probability; this is similar to the ZnO–In_2_S_3_ rod heterostructure system, which also exhibits higher photocatalytic properties than those of its ZnO counterpart [30]. Furthermore, the higher surface area of the ZnO–Sn_2_S_3_ nanorods exposed to organic dyes can provide more active catalytic sites to increase photocatalytic activity [31]. The aforementioned factors explain the markedly improved photocatalytic performance of the ZnO nanorods coated with the Sn_2_S_3_ shell layers in this study. To confirm the recycling ability and long-term chemical stability of the ZnO–Sn_2_S_3_ nanorods, a photodegradation test of MB solution containing ZnO–Sn_2_S_3_ nanorods under light irradiation was repeated three times as exhibited in Fig. 5f. No significant variation was found in the photodegradation efficiency of the ZnO–Sn_2_S_3_ nanorods in different test runs. It has been shown that the reaction of the photogenerated holes with the surface oxygen of ZnO is the main reason for the photocorrosion process of ZnO. The suppression of the reaction between the photogenerated holes and surface oxygen is beneficial to stabilize the photoactivity of ZnO [32]. The high photoactivity and photostability for the ZnO–Sn_2_S_3_ nanorods in this study is associated with the ZnO nanorods capsulated by sheet-like Sn_2_S_3_ crystallites. This engendered more MB molecules could be adsorbed, which consumed more holes photoexcited from ZnO core and competed with the dissolution process of ZnO. This finding demonstrates that these ZnO–Sn_2_S_3_ nanorods are stable and reusable catalysts for photodegrading MB organic dyes.

**Fig. 6:**
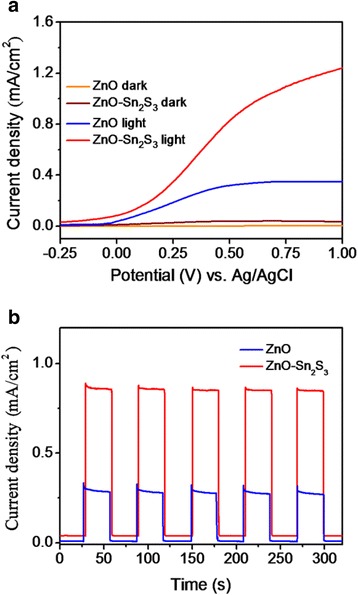
**a** Current density vs. potential curves for various nanorod samples with and without light illumination. **b** Cyclic current density vs. time curves for various nanorod samples under chopped light illumination

Figure 6a shows the photocurrent density vs. the potential curves of the ZnO and ZnO–Sn_2_S_3_ nanorods with and without light illumination. Under light irradiation, the measured photocurrent densities of the ZnO and ZnO–Sn_2_S_3_ nanorods were approximately 0.32 and 0.84 mA cm^−2^ at 0.5 V, respectively. The ZnO nanorod sample yielded relatively low photocurrent under light illumination. However, the sequential combination of the Sn_2_S_3_ shell layers onto the surfaces of the ZnO nanorods significantly enhanced the photocurrent density. These results confirmed that the ZnO–Sn_2_S_3_ nanorods exhibited efficient visible light absorption ability and excellent interfacial charge transformation. Figure 6b displays the photocurrent responses of the ZnO and ZnO–Sn_2_S_3_ nanorods at an applied potential of 0.5 V. The ZnO–Sn_2_S_3_ nanorods exhibited steady and highly repeatable photocurrent responses during on–off cycles of light illumination. Notably, photoexcited electrons in the Sn_2_S_3_ are injected into ZnO because of the band alignment of the heterostructure, as discussed earlier in the present text. This is attributed to the type II band alignment between the ZnO and Sn_2_S_3_; the effective photoexcited charge separation has been widely reported in other heterostructure systems [33, 34]. The aligned ZnO nanorods provide a conduction path, and numerous photoexcited electrons are transferred from Sn_2_S_3_ and ZnO to the F-doped SnO_2_ electrode and are then finally transferred to the platinum electrode. After the photogenerated carriers are transferred rapidly in the PEC system, the electrons travel through F-doped SnO_2_ to the platinum electrode and react with the electrolyte, yielding a reduction reaction, whereas the holes in the valence band of Sn_2_S_3_ react with the electrolyte, yielding an oxidation reaction [34]. Consequently, the ZnO nanorods coated with the Sn_2_S_3_ shell layers exhibit excellent PEC activity compared with that of pure ZnO rods. In this study, the superior PEC performance of the ZnO–Sn_2_S_3_ nanorod heterostructures is attributable to the increased contact area between the nanorods and adsorbed electrolyte molecules resulting from the unique three-dimensional sheet-like Sn_2_S_3_ layers of the ZnO–Sn_2_S_3_ rod heterostructures. Furthermore, Sn_2_S_3_ exhibits superior optical absorption ability, providing high visible light-harvesting efficiency. These factors account for the superior PEC activity of the ZnO–Sn_2_S_3_ nanorods in this study.

## Conclusions

In this study, ZnO–Sn_2_S_3_ core–shell nanorod heterostructures were synthesized by sputtering Sn_2_S_3_ shell layers onto hydrothermally derived ZnO nanorods. The microstructural analyses revealed that the surfaces of the ZnO–Sn_2_S_3_ nanorod heterostructures had a sheet-like texture, and the nanorod heterostructures were highly crystalline. The optical absorption spectra analysis revealed that the ZnO–Sn_2_S_3_ nanorods exhibited a visible light-sensitive feature. Compared with ZnO nanorods, the ZnO–Sn_2_S_3_ nanorods exhibited enhanced photodegradation efficiency of MB organic dyes and improved PEC performance under light illumination. The unique sheet-like shell structures resulted in a high surface area of the nanorod heterostructures. Moreover, the suitable band alignment between the ZnO and Sn_2_S_3_ decreased the recombination efficiency of the light-excited carriers in the composite nanorods. These factors explain the superior photocatalytic and PEC properties of the ZnO–Sn_2_S_3_ nanorod heterostructures.

## Abbreviations

HRTEM: High-resolution transmission electron microscopy
MB: Methylene blue
PEC: Photoelectrochemical
PL: Photoluminescence
SEM: Scanning electron microscopy
XRD: X-ray diffraction
